# Prognostic value of baseline circulating tumor DNA levels in metastatic castration-resistant prostate cancer: a systematic review and meta-analysis

**DOI:** 10.3389/fimmu.2026.1691229

**Published:** 2026-03-04

**Authors:** Yonghe Liao, Yuxuan Lin, Bo Luo, Jinhai Shen

**Affiliations:** 1 Department of Pharmacy, The Second Affiliated Hospital of Guangxi Medical University, Nanning, Guangxi, China; 2 Department of Pharmacy, Guangxi Hospital Division of The First Affiliated Hospital, Sun Yat-sen University, Nanning, Guangxi, China; 3 State Key Laboratory of Natural Medicines, China Pharmaceutical University, Nanjing, Jiangsu, China; 4 Center for New Drug Safety Evaluation and Research, China Pharmaceutical University, Nanjing, Jiangsu, China; 5 School of Basic Medicine and Clinical Pharmacy, China Pharmaceutical University, Nanjing, Jiangsu, China

**Keywords:** biomarker, circulating tumor DNA, meta-analysis, metastatic castration-resistant prostate cancer, survival outcomes

## Abstract

**Background:**

Metastatic castration-resistant prostate cancer (mCRPC) remains a clinically aggressive and lethal disease. Circulating tumor DNA (ctDNA), as a minimally invasive biomarker, has shown prognostic utility in several solid tumors. However, its clinical relevance in mCRPC has not been comprehensively elucidated.

**Methods:**

A systematic search of PubMed and EMBASE was conducted from inception to July 2025 to identify studies evaluating the prognostic impact of baseline ctDNA levels in patients with mCRPC. Eligible studies reported associations between ctDNA levels and survival outcomes. Pooled hazard ratios (HRs) and 95% confidence intervals (CIs) were calculated for overall survival (OS), progression-free survival (PFS), radiographic PFS (rPFS), and prostate specific antigen PFS (PSA-PFS) using random-effects models.

**Results:**

Twenty-four studies encompassing 5,272 patients met the inclusion criteria. Elevated baseline ctDNA levels were significantly associated with inferior OS (HR: 3.45; 95% CI: 2.77–4.31), PFS (HR: 2.26; 95% CI: 1.74–2.93), rPFS (HR: 2.39; 95% CI: 1.85–3.10), and PSA-PFS (HR: 2.50; 95% CI: 1.81–3.46). Subgroup analyses showed that the negative prognostic impact of high baseline ctDNA levels on OS remained consistent regardless of detection methods, treatment types, and stratification strategies.

**Conclusion:**

High baseline ctDNA levels—regardless of measurement approach or therapeutic context—are associated with markedly worse clinical outcomes in mCRPC. These findings highlight ctDNA as a clinically meaningful, noninvasive prognostic biomarker, supporting its integration into personalized risk stratification frameworks and therapeutic decision-making in mCRPC.

**Systematic Review Registration:**

https://www.crd.york.ac.uk/prospero/, identifier CRD420251108650.

## Introduction

Metastatic castration-resistant prostate cancer (mCRPC) represents the most advanced and fatal stage of prostate cancer, defined by disease progression despite effective androgen deprivation therapy (1). Although therapeutic advances—including androgen receptor signaling inhibitors (ARSIs), taxane-based chemotherapy, radioligand therapy, and targeted agents—have modestly extended survival for subsets of patients, overall prognosis remains poor and clinical trajectories are highly variable (1). Current prognostic assessment relies heavily on prostate-specific antigen (PSA) and conventional imaging, both of which have notable limitations. PSA lacks specificity and does not always reflect true tumor burden or biological aggressiveness, while imaging modalities are insensitive to early metastatic spread and offer little insight into tumor molecular dynamics (2, 3). These limitations underscore the urgent need for robust, minimally invasive biomarkers capable of improving risk stratification, guiding treatment selection, and informing clinical trial design in mCRPC.

Circulating tumor DNA (ctDNA)—genomic fragments released into the bloodstream by apoptotic and necrotic tumor cells—has emerged as a promising liquid biopsy analyte offering key advantages. It provides a real-time snapshot of tumor burden, captures spatial and temporal heterogeneity, and enables dynamic monitoring of molecular evolution under therapeutic pressure (4–6).

Various ctDNA assay approaches exist and differ in analytic sensitivity and clinical interpretability. Broadly, assays can be categorized as tumor-informed, which require sequencing of matched tumor tissue to identify patient-specific mutations, and tumor-agnostic, which rely on predefined gene panels without prior tumor profiling (7). ctDNA can also be measured quantitatively (e.g., tumor fraction) or assessed qualitatively (e.g., detectable *vs*. undetectable alterations) (8). These methodological distinctions have important clinical implications. For example, in bladder cancer, quantitative ctDNA/MRD assays have become central to minimally invasive risk stratification and disease surveillance (9). This context underscores how assay design may shape clinical applicability and helps frame the heterogeneity of ctDNA methodologies observed across studies in mCRPC.

In malignancies such as colorectal, lung, and breast cancer, elevated baseline ctDNA levels have consistently been linked to inferior survival outcomes (10–12). Similarly, in mCRPC, observational evidence increasingly supports high baseline ctDNA levels as a marker of adverse clinical outcomes (13). However, the magnitude and consistency of this association vary across studies, likely due to heterogeneity in study designs, ctDNA detection platforms, threshold definitions, and patient characteristics. Notably, no comprehensive meta-analysis has quantitatively synthesized this evidence.

Considering this background, we conducted a systematic review and meta-analysis to quantitatively assess the association between baseline ctDNA levels and key survival endpoints in mCRPC. In addition, we examined methodological and clinical factors that might contribute to variability in reported findings. This study aims to clarify the prognostic significance of baseline ctDNA levels and to evaluate its potential integration into risk stratification frameworks and therapeutic decision-making, thereby informing more individualized management strategies for this aggressive disease. 

## Methods

### Protocol and guideline

This meta-analysis was prospectively registered in the International Prospective Register of Systematic Reviews (registration ID: CRD420251108650) and conducted in accordance with the Preferred Reporting Items for Systematic Reviews and Meta-Analyses (PRISMA) 2020 reporting guidelines (14).

### Data sources and search strategy

PubMed and Embase were comprehensively searched from database inception to July 2025 to identify all relevant studies. The search strategy combined terms related to ‘*circulating tumor DNA*’ and ‘*metastatic castration-resistant prostate cancer*.’ A detailed list of the complete search strategies, including all search terms and retrieval counts, is provided in **Supplementary Table 1**.

### Selection criteria

All study designs examining the association between baseline ctDNA levels and prognosis in mCRPC were eligible for inclusion. These encompassed prospective cohort studies (with baseline ctDNA sampling), retrospective cohort studies (using real-world clinical–genomic databases), and biomarker sub-studies of randomized controlled trials (RCTs) reporting mCRPC-specific data. In all eligible studies, “baseline ctDNA levels” was operationally defined as blood samples obtained immediately prior to initiation of the specific therapy under investigation in each included study, regardless of treatment line within the mCRPC setting. Where reported, sampling was performed within protocol-defined windows before treatment initiation. *In vitro* experiments, animal studies, and case reports were excluded, as they do not directly inform clinical prognostic utility in human mCRPC. Studies with insufficient mCRPC-specific data (e.g., pooled analyses with other prostate cancer stages without stratified reporting) or those published in languages other than English were also excluded.

### Data extraction and quality assessment

Data extraction was performed by a single investigator and independently verified by a second reviewer. When both adjusted and unadjusted estimates were available, adjusted data were preferentially included. Extracted variables included the first author’s name, year of publication, study design, treatment regimen, baseline ctDNA detection method, cut-off definition for high/positive ctDNA levels, and hazard ratios (HRs) with corresponding 95% confidence intervals (CIs) for reported survival outcomes. Study quality was independently assessed by two authors using the Quality in Prognosis Studies (QUIPS) tool (15).

### Statistical analysis

The prognostic significance of baseline ctDNA levels was assessed for overall survival (OS), progression-free survival (PFS), radiographic PFS (rPFS), and prostate-specific antigen PFS (PSA-PFS) in patients with mCRPC. Pooled estimates for each endpoint were obtained by synthesizing HRs with their corresponding 95% CIs. Statistical heterogeneity was quantified using the *I^2^* statistic and Cochran’s *Q* test. When *I^2^* exceeded 50% and the *p*-value was less than 0.10, indicating substantial heterogeneity, a random-effects model was applied; otherwise, a fixed-effects model was used. Publication bias was assessed using funnel plots and Egger’s regression test. Sensitivity analyses were performed by sequentially omitting one study at a time to evaluate the robustness of the pooled results. All statistical analyses were conducted in R software (version 4.5.1), with statistical significance set at *p* < 0.05.

## Results

### Study selection

The initial search retrieved 1,580 records. After removing duplicates, 1,260 unique studies remained for preliminary screening. Following title and abstract review, 1,215 studies were excluded as irrelevant, leaving 45 articles for full-text assessment. Ultimately, 24 studies met all eligibility criteria and were included in the meta-analysis (16–39). The study selection process is illustrated in **Supplementary Figure 1**.

### Characteristics of the included studies

A total of 24 studies published between 2018 and 2025, encompassing 5,272 patients with mCRPC, were included in this meta-analysis. The majority were prospective cohort studies (*n* = 8) and retrospective cohort analyses (*n* = 11), supplemented by prespecified exploratory biomarker analyses from randomized phase 2/3 trials (*n* = 5). Sample sizes ranged from 37 to 776 patients (median: 165). Treatments included ARSI (enzalutamide, abiraterone), chemotherapy (cabazitaxel), radiopharmaceuticals (^177^Lu-PSMA-617), and combinations (e.g., enzalutamide with atezolizumab). Baseline ctDNA levels measurements reflect heterogeneous treatment contexts, spanning first-line to later-line mCRPC therapies. ctDNA detection primarily relied on NGS, including commercial panels (Guardant360), custom panels (AR-ctDETECT), and combined methods (targeted NGS + ddPCR). Definitions of high/positive ctDNA levels varied, including ctDNA% ≥0.180, >30%, detectable ctDNA, or >1 genomic alteration. Comparison groups were classified as high *versus* low ctDNA levels or positive *versus* negative ctDNA. Survival outcomes included OS, rPFS, PFS, and PSA-PFS, with HR and 95% CI reported for each. The characteristics of the included studies are summarized in **Table 1**.

**Table 1:** Characteristics of the included studies.

Studies	Study design	Country	Sample size	Treatment	Treatment line	ctDNA detection method	Cut-off for positive/high ctDNA value	Comparison groups	Survival outcomes reported
Shaya et al. (16)	Retrospective cohort study	USA	63	Mixed (63.5% ARSI, 34.9% chemotherapy, 15.9% radium, etc.)	Mixed lines of therapy (median 1 line, range 0-5)	Commercial NGS (Guardant360: 85.7%, Tempus xF: 14.3%)	>1 alteration	>1 alteration vs. ≤1 alteration	OS
Knutson et al. (17)	*Post-hoc* biomarker analysis of a phase 3 trial	Multinational	776	Enzalutamide with or without abiraterone	First-line	Custom NGS (AR-ctDETECT)	ctDNA aneuploidy fraction >14.2%	ctDNA+ vs. ctDNA−	rPFS, OS
Bono et al., 2025 (18)	Prespecified exploratory biomarker analysis within a phase 3 trial	Multinational	173	^177^Lu-PSMA-617 (n = 82), ARSI (n = 91)	Second-line (progression on 1 prior ARPI)	In-house custom ctDNA panel	Continuous variable (HR per 1-unit increase in ctDNA%)	Per 1-unit increase in ctDNA%	rPFS, OS
Conteduca et al. (19)	Prospective cohort study	Italy	220	Abiraterone (n = 140), enzalutamide (n = 80)	Mixed lines (27.3% first-line, 32.3% second-line)	Targeted NGS + ddPCR (AR CN) + Quant-iT PicoGreen DNA Assay	ctDNA% ≥0.180 (median)	High ctDNA (≥0.180) vs. Low ctDNA (<0.180)	PSA-PFS, OS
Annala et al. (20)	Prespecified exploratory biomarker analysis within a randomized phase 2 trial	Canada, Australia	95	Cabazitaxel (n = 45), ARPI (abiraterone or enzalutamide; n = 50)	First-line	Deep targeted sequencing of 73 genes in plasma cfDNA to measure ctDNA%	High baseline ctDNA%: ≥15% (above cohort median)	High ctDNA (≥15%) vs. Low ctDNA (<15%)	OS
Goodall et al. (21)	Prespecified exploratory biomarker analysis within a randomized phase 2 trial	Multinational	216	Abiraterone with or without ipatasertib	≥Third-line	Custom 58-gene panel (QIAseq Targeted DNA)	Presence of detectable ctDNA	Detectable ctDNA vs. undetectable ctDNA	rPFS
Sweeney et al. (22)	Prespecified exploratory biomarker analysis within a randomized phase 3 trial	Multinational	408	Enzalutamide with or without atezolizumab	Post-abiraterone progression (≥2nd-line)	FoundationOne Monitor (NGS-based)	Detectable ctDNA TF	Detectable TF vs. undetectable TF	rPFS OS
Maurice-Dror et al. (23)	Prospective cohort study	Canada	410	ARSI (90%)	First-line	Deep targeted sequencing of plasma cfDNA	ctDNA% >30%	High ctDNA% vs. Low ctDNA%	PSA-PFS, OS
Tolmeijer et al. (24)	Prespecified exploratory biomarker analysis within a phase Ib trial	Multinational	37	LuPSMA plus pembrolizumab	Not specified	Targeted sequencing of plasma cfDNA	ctDNA% >30%	High ctDNA% vs. Low ctDNA%	rPFS
Kohli et al. (25)	Prospective longitudinal cohort study	Multinational	144	Mixed	First-line (87% ARPI-naive)	Targeted NGS (120-gene PredicineLDT panel) + Quant-iT PicoGreen DNA Assay	Above the median ctDNA%	High ctDNA% (≥ median) vs. Low ctDNA% (< median)	OS
Mizuno et al. (26)	Prospective cohort study	Japan	100	Abiraterone (n = 51) or enzalutamide (n = 49)	Mixed (13% prior ARPI)	Targeted NGS (88-gene panel, eVIDENCE)	ctDNA% ≥0.4%	HRR defect (ATM/BRCA2) vs. no defect TP53 defect vs. no defect	PFS, OS
Tolmeijer et al. (27)	Prospective observational study	Netherlands Canada	81	First-line ARSI (abiraterone: n =5 1; enzalutamide: n = 30)	First-line	Targeted sequencing (73-gene panel) + genome-wide CNV/SNP analysis	ctDNA detected (based on sensitivity thresholds of the assay)	Detected ctDNA vs. undetected ctDNA at baseline	PFS, OS
Sumanasuriya et al. (28)	Prespecified exploratory study of two phase 3 trials	Multinational	188	Taxane chemotherapy (docetaxel or cabazitaxel)	Mixed: First-line taxane (FIRSTANA cohort) & Post-docetaxel (PROSELICA cohort)	Low-pass whole-genome sequencing	Continuous variable (log_10_-transformed TF)	Baseline ctDNA TF	rPFS, PSA-PFS, OS
Torquato et al. (29)	Prospective cohort study	USA	62	ARSI (abiraterone: 56.5%; enzalutamide: 40.3%; combo: 3.2%)	Mixed lines	Deep NGS (46-gene panel)	High ctDNA (defined by mutation burden, not a fixed % threshold)	High ctDNA vs. Low ctDNA	PFS, OS
Ruiz-Vico et al., 2025 (30)	Prespecified exploratory biomarker analysis within a phase 3b trial	Multinational	157	Docetaxel with or without enzalutamide	≥ Second-line	Custom NGS panel (PCF_SELECT)	Detectable (positive if ctDNA was detected in plasma)	ctDNA+ vs. ctDNA–	PFS
Kwan et al. (31)	*Post-hoc* biomarker analysis of a randomized phase 2 trial	Multinational	178	^177^Lu-PSMA-617 (n = 96), cabazitaxel (n = 82)	Second-line (post-docetaxel)	Targeted sequencing (cfDNA + matched WBC DNA)	ctDNA%: <2%, 2-30%, >30%	ctDNA% categories within treatment arm	PFS, OS
Khalaf et al. (32)	Prespecified exploratory analysis within a phase 2 trial	Multinational	202	Abiraterone plus prednisone, enzalutamide	First-line and second-line	Deep-targeted sequencing of ctDNA	ctDNA ≥2%	High ctDNA (≥2%) vs. Low ctDNA (<2%)	OS
Stover et al. (33)	Real-world observational study	USA	78	Mixed lines of therapy	Mixed lines (19% 1st line, 24% 2nd line, 22% 3rd line, and 35% 4th line)	FoundationOne Liquid (commercial CGP assay)	cTF ≥10%	High cTF (≥10%) vs. Low cTF (<10%)	OS
Jayaram et al. (34)	Prespecified exploratory biomarker analysis of a phase 2 trial	Multinational	128	Abiraterone acetate plus glucocorticoids	First-line (chemotherapy-naive)	Custom targeted NGS + rolling B-allele method	Detectable	ctDNA detected vs. not detected	PFS, OS
Fonseca et al. (35)	Metacohort analysis of two phase 2 trials	Multinational	463	First-line ARSI (91% abiraterone or enzalutamide), taxanes (8%)	Mixed lines (first- to third-line)	Deep targeted sequencing (73-gene panel) + matched WBC	High ctDNA%: >30% Low ctDNA%: 2-30% Undetectable ctDNA: <2%	High ctDNA (>30%) vs. low ctDNA (2-30%) vs. undetectable ctDNA (<2%)	PSA-PFS, OS
Nørgaard et al. (36)	Prospective cohort study	Denmark	143	First-line ARSI (abiraterone or enzalutamide)	First-line (ARSI-naive)	Low-pass whole-genome sequencing (median 0.5× coverage)	ctDNA%: <3.2%, 3.2-19.9%, >19.9%	High ctDNA (>19.9%) vs. intermediate ctDNA (3.2-19.9%) vs. low ctDNA (<3.2%)	PSA-PFS, PFS, OS
Reichert et al., 2023 (37)	Real-world observational cohort study	USA	198	Mixed systemic therapy	Mixed lines	FoundationOne® Liquid/Liquid CDx (NGS)	TF ≥10%	TF ≥10% vs. TF <10%	OS
Pan et al. (38)	Prospective cohort study	China	74	Abiraterone	Mixed (57.5% first-line)	Targeted NGS (15-gene HRR panel)	ctDNA%: >2%	ctDNA% >2% vs. ≤2%	PFS
Azad et al. (39)	*Post-hoc* biomarker analysis of a phase 3 trial	Multinational	678	Talazoparib plus enzalutamide or placebo plus enzalutamide	First-line	FoundationOne Liquid CDx (plasma TF via aneuploidy)	Quantifiable aneuploidy	High vs. Low baseline ctDNA burden	rPFS

ctDNA, circulating tumor DNA; ARPI, androgen receptor pathway inhibitor; HR, hazard ratio; CI, confidence interval; rPFS, radiographic progression-free survival; PSA-PFS, prostate-specific antigen progression-free survival; PFS, progression-free survival; NGS, next-generation sequencing; ddPCR, droplet digital PCR; cfDNA, cell-free DNA; WBC, white blood cell; CNV, copy number variation; SNP, single nucleotide polymorphism; WES, whole exome sequencing; sWGS, shallow whole genome sequencing; HRR, homologous recombination repair; ARSI, androgen receptor signaling inhibitor; PSMA, prostate-specific membrane antigen; ADT, androgen deprivation therapy; cTF, composite TF; TF, DNA%; ctDNA%, circulating tumor DNA fraction.

According to the QUIPS assessment, the overall methodological quality of the included studies was moderate to high. Most studies showed low risk of bias in outcome measurement, confounding control, and statistical reporting, while a minority demonstrated moderate risk in participation and attrition domains. Full domain-level evaluations for each study are provided in **Supplementary Table 2**.

### Baseline ctDNA levels and prognosis in mCRPC

**Figure 1:**
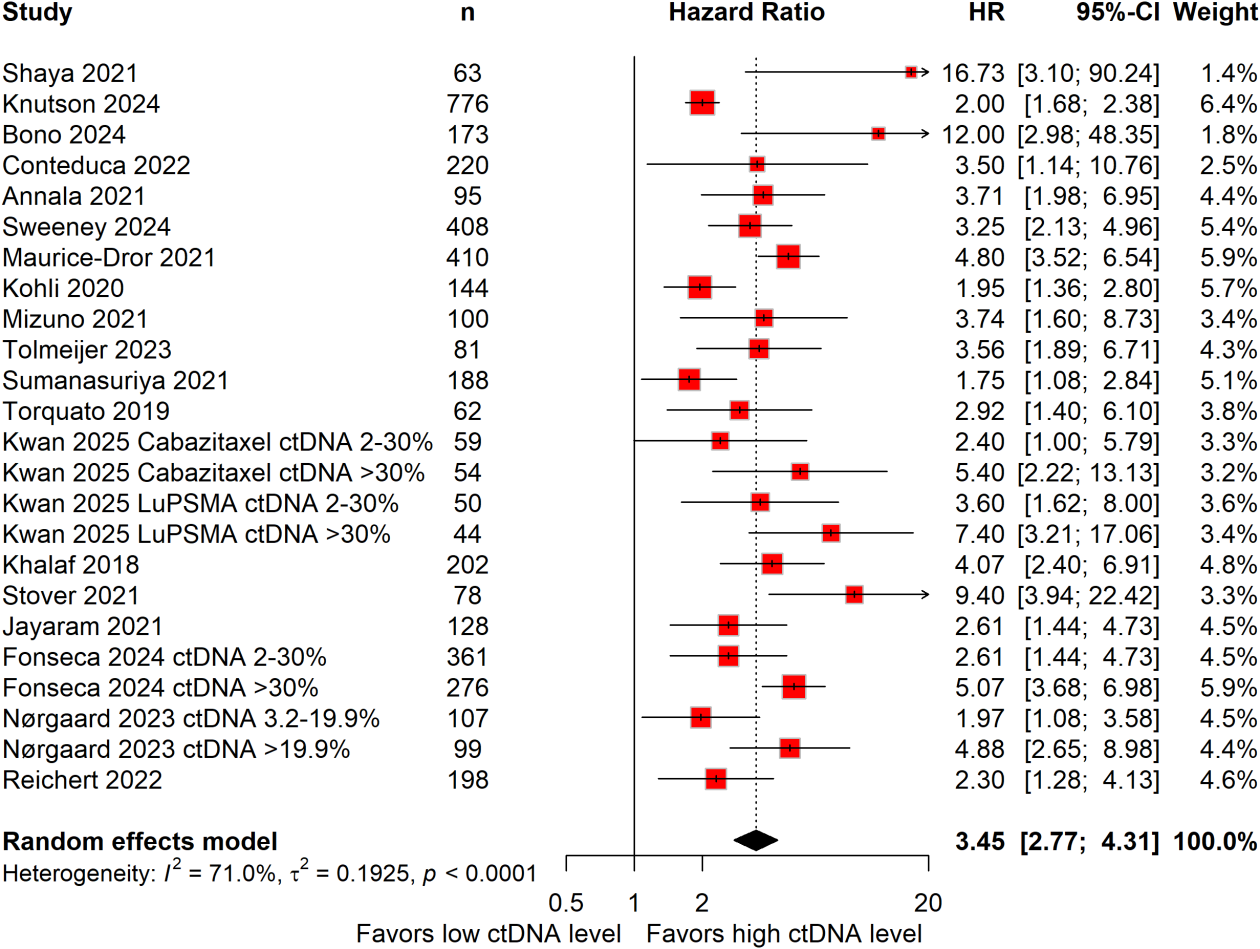
Forest plots demonstrating the association between baseline circulating tumor DNA levels and overall survival in patients with metastatic castration-resistant prostate cancer.

Nineteen studies, encompassing 4,110 patients, evaluated the prognostic significance of baseline ctDNA levels for OS in mCRPC (16–20, 22, 23, 25–29, 32–37). Given the substantial heterogeneity across studies (*I^2^* = 71%), a random-effects model was used to generate the pooled estimate. High baseline ctDNA levels were associated with a 245% higher risk of mortality (HR: 3.45; 95%CI: 2.77–4.31; **Figure 1**), underscoring their strong negative prognostic value in this population.

**Figure 2:**
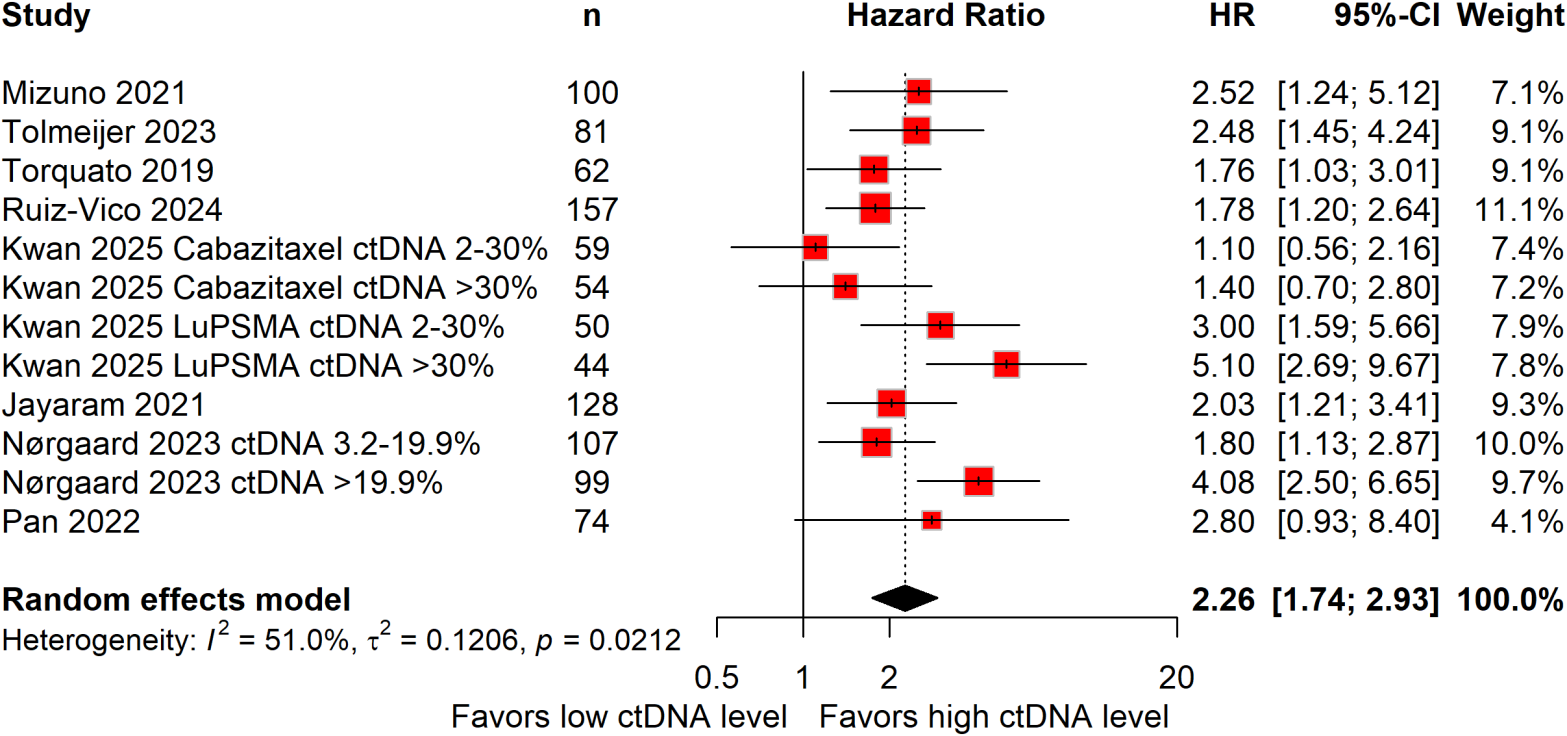
Forest plots demonstrating the association between baseline circulating tumor DNA levels and progression-free survival in patients with metastatic castration-resistant prostate cancer.

Eight studies, including 2,395 patients, assessed PFS (26, 27, 29–31, 34, 36, 38). Elevated baseline ctDNA levels were associated with a 126% higher risk of disease progression compared with low ctDNA levels (HR: 2.26; 95%CI: 1.74–2.93; **Figure 2**), with moderate heterogeneity observed (*I^2^* = 51%).

**Figure 3:**
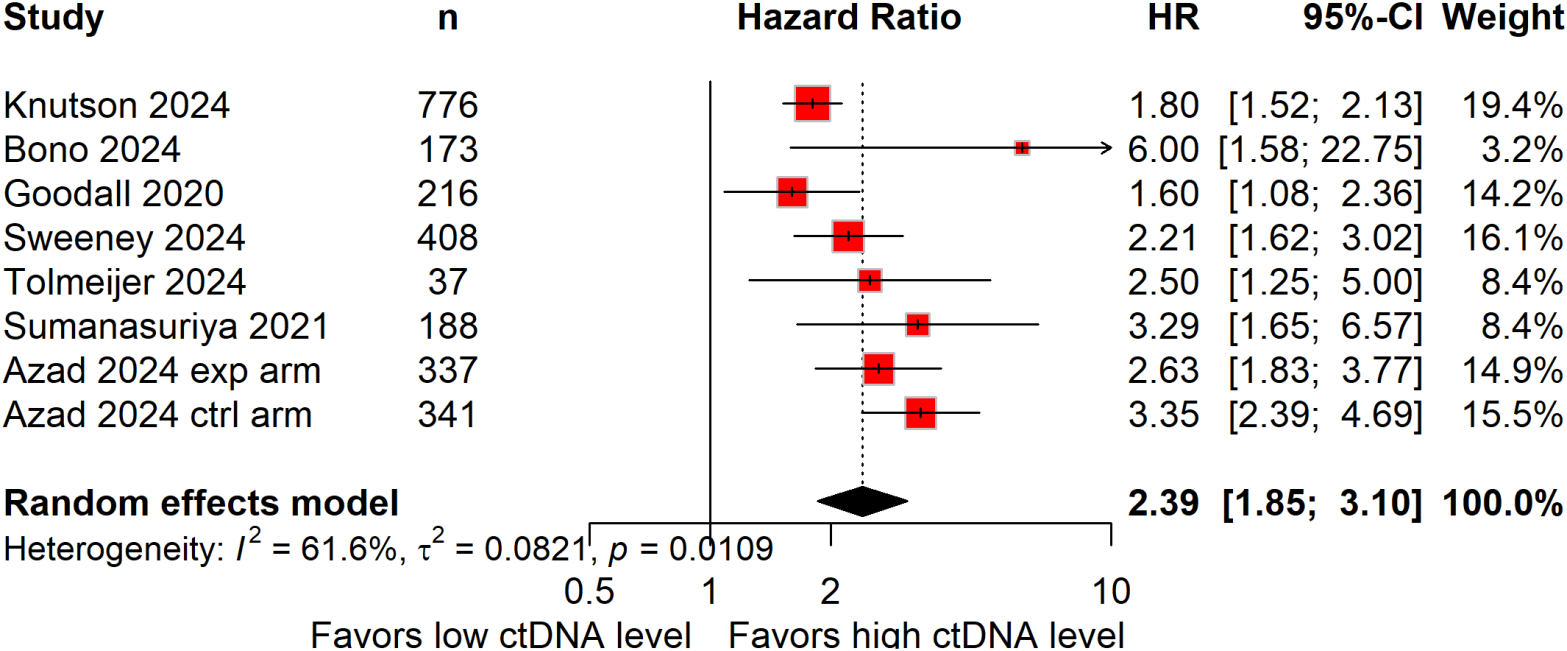
Forest plots demonstrating the association between baseline circulating tumor DNA levels and radiographic progression-free survival in patients with metastatic castration-resistant prostate cancer.

Seven studies, totaling 2,476 patients, evaluated rPFS (17, 18, 21, 22, 24, 28, 39). High baseline ctDNA levels were associated with a 139% increased risk of radiographic progression (HR: 2.39; 95%CI: 1.85–3.10; **Figure 3**), with significant heterogeneity present (*I^2^* = 61.6%).

**Figure 4:**
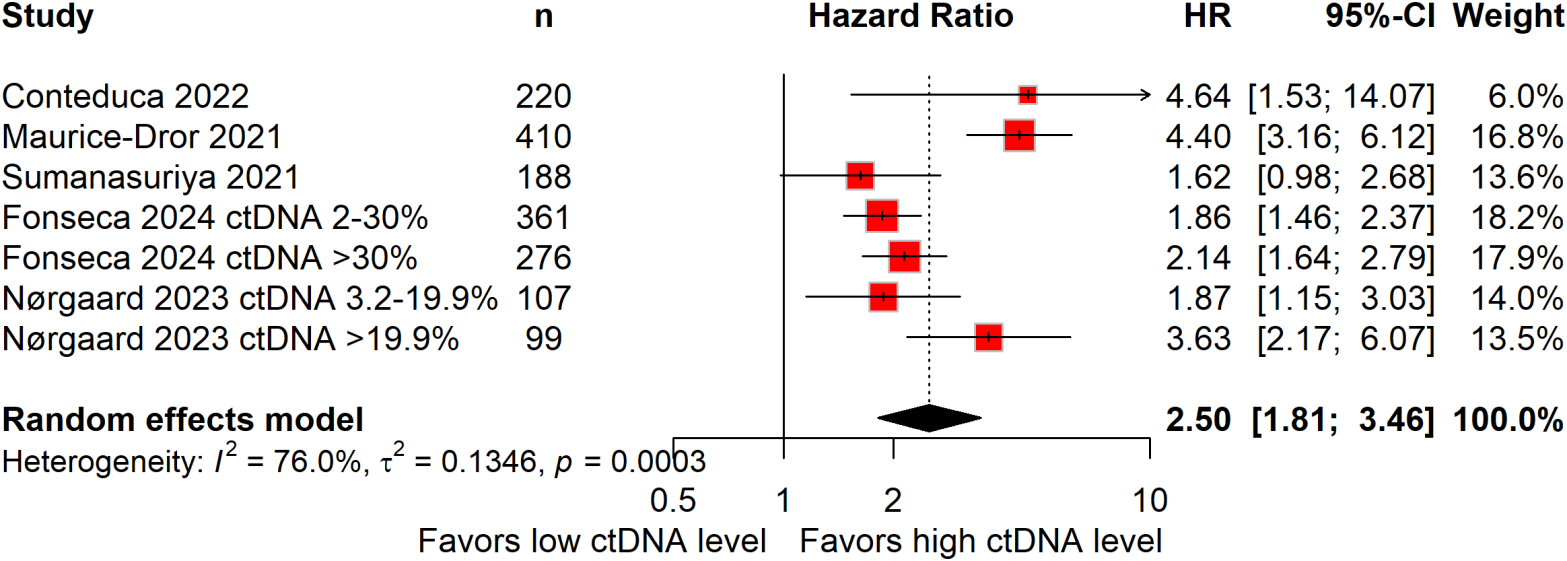
Forest plots demonstrating the association between baseline circulating tumor DNA levels and prostate specific antigen progression-free survival in patients with metastatic castration-resistant prostate cancer.

Five studies, involving 1,661 patients, examined PSA-PFS (19, 23, 28, 35, 36). Elevated baseline ctDNA levels predicted a 150% higher risk of PSA progression (HR: 2.50; 95%CI: 1.81–3.46; **Figure 4**), with significant heterogeneity detected (*I^2^* = 76.0%).

### Subgroup analysis

To further assess the prognostic significance of ctDNA levels in mCRPC, subgroup analyses were conducted according to ctDNA levels detection method, treatment regimen, definition of high/positive ctDNA levels, and study design. This stratified approach allowed exploration of potential variations in the prognostic effect of ctDNA levels across methodological and clinical contexts, thereby enhancing our understanding of its role as a prognostic biomarker in mCRPC.

Based on detection method, the cohort was categorized into four subgroups: commercial next-generation sequencing (NGS), custom NGS, combined detection methods, and shallow/low-pass whole-genome sequencing (sWGS/lpWGS). Across all detection methods, elevated baseline ctDNA levels were consistently associated with significantly worse OS (**Supplementary Figure 2**).

When stratified by treatment regimen, high baseline ctDNA levels were associated with shorter OS in patients receiving ARSIs (HR: 3.16; 95% CI: 2.48–4.02), taxane chemotherapy (HR: 2.63; 95% CI: 1.35–5.11), and lutetium-177–PSMA radioligand therapy (HR: 5.11; 95% CI: 2.44–10.71) (**Supplementary Figure 3**).

When categorized by definition of high/positive ctDNA levels—either based on ctDNA percentage thresholds or dichotomous detectability—elevated ctDNA levels were associated with significantly shorter OS in both groups (**Supplementary Figure 4**).

Finally, stratification by study design demonstrated that the detrimental prognostic association of high baseline ctDNA levels with OS persisted across both prospective and retrospective studies (**Supplementary Figure 5**).

### Publication bias and sensitivity analysis

Funnel plots and Egger’s test indicated publication bias for OS (*P* = 0.0133; **Supplementary Figure 6A**) but not PFS (*P* = 0.7152; **Supplementary Figure 6B**). Sensitivity analyses confirmed robustness; no single study significantly altered pooled HRs for OS or PFS (**Supplementary Figure 7**).

## Discussion

This meta-analysis, encompassing 24 studies with 5,272 patients with mCRPC, provides compelling evidence that elevated baseline ctDNA levels are strongly and consistently associated with inferior survival outcomes. Across key endpoints—OS, PFS, rPFS, and PSA-PFS—high baseline ctDNA levels conferred a 245% increased mortality risk (HR: 3.45; 95% CI: 2.77–4.31) and 2.26- to 2.50-fold higher risks of disease progression. This prognostic association persisted across diverse detection methods, therapeutic modalities, and threshold definitions, supporting ctDNA levels as a robust, reproducible biomarker for risk stratification in mCRPC.

The biological plausibility of these findings is well grounded. ctDNA represents tumor-derived genomic fragments released into the circulation via apoptosis, necrosis, or active secretion (40). Its abundance reflects tumor burden, cellular turnover, and metastatic spread, while also capturing genomic instability and clonal heterogeneity—hallmarks of aggressive disease (4, 41, 42). In mCRPC, high ctDNA levels often co-occur with adverse genomic alterations such as *AR* amplification, *TP53* and *PTEN* loss, and homologous recombination repair (HRR) defects (17, 26, 43, 44). These aberrations drive treatment resistance and aggressive clinical trajectories, providing a mechanistic rationale for the observed association between elevated ctDNA levels and poor prognosis (17).

Importantly, the scope of this meta-analysis was intentionally confined to baseline ctDNA levels rather than ctDNA genotyping. While ctDNA-based molecular profiling—including detection of homologous recombination repair alterations, AR pathway aberrations, or microsatellite instability/tumor mutation burden status—has become central to precision therapy selection in mCRPC, such analyses require assay-specific mutation-level data that were heterogeneously reported and not amenable to quantitative synthesis across studies. In contrast, ctDNA levels represent a platform-agnostic metric shared across sequencing approaches and clinical trial designs, enabling robust meta-analytic evaluation. As such, our findings position baseline ctDNA levels as a foundational prognostic layer upon which genotypic and dynamic ctDNA analyses may be integrated in future biomarker-driven frameworks.

Although PSA remains a widely used and cost-effective cornerstone of mCRPC management, our synthesis indicates that baseline ctDNA levels offer complementary prognostic insight grounded in distinct biology. While ctDNA levels often correlate with PSA—reflecting their shared association with overall disease burden—its prognostic effect remained strong and independent across studies. The magnitude of the pooled HR for elevated ctDNA levels (e.g., HR = 3.45 for OS) suggests that they may capture a more direct measure of tumor genomic burden and molecular aggressiveness. Notably, the less common but clinically informative discordant phenotype of high ctDNA levels with low PSA may indicate PSA-indifferent or aggressive variants in which PSA underestimates disease activity (16, 18, 20, 22, 24). Therefore, ctDNA levels should not be viewed as a replacement for PSA but rather as a complementary biomarker that, when integrated with PSA and established clinical variables, could refine baseline risk stratification and enhance patient assessment.

Our subgroup analyses further support the prognostic relevance of baseline ctDNA levels across treatment modalities. Elevated ctDNA levels were consistently associated with inferior OS in patients treated with ARSIs, taxane chemotherapy, and ^177^Lu-PSMA-617 radioligand therapy. The magnitude of the association appeared greatest in the radioligand therapy subgroup (HR: 5.11), which may reflect a subset of biologically aggressive tumors characterized by higher disease burden and heterogeneous or reduced PSMA expression. Importantly, these findings are based on baseline measurements and do not account for treatment-specific molecular dynamics. Collectively, the consistency of the association across distinct therapeutic classes is more compatible with ctDNA levels reflecting underlying disease aggressiveness rather than serving as a therapy-specific predictive marker. Clinically, this supports the potential role of baseline ctDNA levels as a general risk-stratification tool at treatment initiation, irrespective of initial therapy selection.

Importantly, the prognostic association was consistent across detection platforms—including commercial NGS panels, custom assays, combined NGS/digital droplet PCR approaches, and shallow or low-pass whole-genome sequencing. This methodological resilience suggests that clinically relevant thresholds can be achieved across laboratories, although standardization of pre-analytical handling, assay sensitivity, and bioinformatic pipelines remains essential. Current heterogeneity in definitions of “high” ctDNA levels—ranging from detectability to quantitative tumor fraction cut-offs (e.g., ≥0.180 or >30%)—limits cross-study comparability and clinical adoption. Consensus guidelines to harmonize ctDNA quantification and reporting will be critical for regulatory validation and routine implementation.

From a clinical trial perspective, baseline ctDNA levels could enhance patient selection, stratification, and endpoint sensitivity. Incorporating ctDNA levels into eligibility or stratification criteria may balance baseline risk across arms, while serial monitoring could detect molecular progression earlier than imaging or PSA. Early rises in ctDNA levels have been shown to precede radiographic progression by weeks to months in mCRPC, offering a window for therapeutic adaptation (22, 35, 45). Future biomarker-driven trials should assess whether ctDNA-guided escalation, switching, or de-escalation strategies improve survival and quality of life.

In addition to its value for clinical trial design, baseline ctDNA levels also hold promise for integration into clinical risk-stratification frameworks. Future prognostic models could incorporate ctDNA fraction together with established clinical variables such as lactate dehydrogenase, albumin, visceral metastases, and ECOG performance status to generate composite risk scores. Such multifactorial models may improve the identification of patients with biologically aggressive disease and support more personalized treatment decisions, including early intensification strategies or allocation to biomarker-enriched therapeutic pathways. As standardized thresholds and assay methodologies are refined, ctDNA-based risk stratification may complement PSA and imaging to guide real-world clinical management in mCRPC.

Several limitations should be acknowledged. First, between-study heterogeneity was substantial, primarily reflecting methodological differences in assay platforms and ctDNA levels threshold definitions. Second, clinical heterogeneity across the included cohorts represents another key source of variability. Differences in prior lines of therapy (e.g., number and type of prior systemic treatments) and extent of metastatic disease (e.g., visceral versus bone-predominant involvement) may influence both ctDNA shedding and survival outcomes. Patients with more heavily pretreated disease or higher metastatic burden often present with elevated ctDNA levels and poorer prognosis, which may contribute to variation in effect estimates across studies. Additional factors—including prior cytotoxic chemotherapy, which can acutely elevate ctDNA levels through tumor cell death independent of long-term prognosis—baseline performance status, and underlying tumor genomic complexity may also confound the observed associations and modify the prognostic effect of baseline ctDNA levels. Third, despite robust sensitivity analyses, the presence of publication bias for OS suggests potential underreporting of null findings. This bias may have led to a modest overestimation of the true effect size. However, the consistency of results across multiple sensitivity and subgroup analyses indicates that the overall conclusion regarding the adverse prognostic impact of elevated baseline ctDNA levels remains robust. Fourth, the observational nature of most included studies and the limited availability of longitudinal ctDNA levels assessments restrict insights into dynamic molecular changes during therapy. Finally, the predominance of data from high-resource settings may constrain generalizability to more resource-limited environments.

Future research should prioritize three complementary directions. First, prospective validation of standardized ctDNA levels thresholds in large, diverse mCRPC cohorts is needed, ideally integrating baseline ctDNA levels into composite prognostic models alongside established clinical and biochemical variables, as well as molecular features derived from ctDNA genotyping where available. Second, interventional trials should evaluate ctDNA-guided treatment adaptation strategies, including whether baseline ctDNA levels in combination with specific genomic alterations (e.g., DNA damage repair defects or AR pathway aberrations) can inform treatment escalation, switching, or de-escalation. Third, future frameworks should integrate ctDNA levels with other liquid biopsy analytes (e.g., circulating tumor cells, extracellular vesicles), advanced molecular profiling, and imaging modalities (e.g., PSMA PET) to develop multimodal, biology-driven risk assessment and treatment personalization strategies in mCRPC.

## Conclusion

This systematic review and meta-analysis demonstrates that elevated baseline ctDNA levels are consistently associated with inferior survival outcomes in patients with mCRPC across multiple therapeutic classes. By quantitatively synthesizing evidence from more than 5,000 patients, our study provides the first comprehensive meta-analytic confirmation of baseline ctDNA levels as a robust, treatment-agnostic prognostic biomarker in mCRPC. Importantly, rather than competing with established biomarkers or ctDNA-based molecular profiling, baseline ctDNA levels should be viewed as a foundational risk-stratification metric that contextualizes clinical variables, PSA, and emerging precision oncology tools. Future studies integrating quantitative, genotypic, and longitudinal ctDNA levels data are warranted to determine whether such multimodal approaches can further refine prognostication and guide treatment personalization in mCRPC.

## Data Availability

The original contributions presented in the study are included in the article/**Supplementary Material**. Further inquiries can be directed to the corresponding author.
