# Supplementary material for: Prognostic value of baseline circulating tumor DNA levels in metastatic castration-resistant prostate cancer: a systematic review and meta-analysis
Source: Front Immunol. 2026 Mar 4;17:1691229. doi: 10.3389/fimmu.2026.1691229 (PMC12996060; doi:10.3389/fimmu.2026.1691229)
Supplement: Supplementary file 1 [file DataSheet1.docx]

**Supplementary Material**

**Prognostic value of baseline circulating tumor DNA levels in metastatic castration-resistant prostate cancer: A systematic review and meta-analysis**

**Supplementary Table 1.** Details of search strategy.

**Supplementary Table 2.** Quality assessment of included studies through the Quality in Prognosis Studies (QUIPS) scale.

**Supplementary Figure 1.** PRISMA flow diagram of study selection.

**Supplementary Figure 2.** Forest plots demonstrating the association between baseline circulating tumor DNA levels and overall survival in patients with metastatic castration-resistant prostate cancer stratified by detection method.

**Supplementary Figure 3.** Forest plots demonstrating the association between baseline circulating tumor DNA levels and overall survival in patients with metastatic castration-resistant prostate cancer stratified by treatment modality.

**Supplementary Figure 4.** Forest plots demonstrating the association between baseline circulating tumor DNA levels and overall survival in patients with metastatic castration-resistant prostate cancer stratified by definition of high/positive ctDNA.

**Supplementary Figure 5.** Forest plots demonstrating the association between baseline circulating tumor DNA levels and overall survival in patients with metastatic castration-resistant prostate cancer stratified by study design.

**Supplementary Figure 6.** Funnel plots and Egger’s tests for overall survival.

**Supplementary Figure 7.** Sensitivity analyses for included studies on overall survival and progression-free survival.

**Supplementary Figure 8.** Sensitivity analyses for included studies on radiographic progression-free survival and prostate specific antigen progression-free survival.

**Supplementary Table 1.** Details of search strategy.

| **Database** | **Search strategy** |
| --- | --- |
| ***PubMed*** | (((("Circulating Tumor DNA"[Mesh]) OR ("Cell-Free Tumor DNA")) OR ("Cell Free Tumor DNA")) OR (ctDNA)) AND ((("Prostatic Neoplasms"[Mesh]) OR ("Prostate Cancer")) OR ("Castration-Resistant Prostate Cancer"))  **Search items: 311** |
| ***Embase*** | ('circulating tumor dna'/exp OR 'cell-free tumor dna' OR 'cell free tumor dna' OR ctdna) AND ('prostatic neoplasms'/exp OR 'prostate cancer' OR 'castration-resistant prostate cancer')  **Search items: 1,269** |

**Supplementary Table 2.** Quality assessment of included studies through the Quality in Prognosis Studies (QUIPS) scale.

| **Study** | **Study Participation** | **Study Attrition** | **Prognostic Factor Measurement** | **Outcome Measurement** | **Confounding Measurement and Account** | **Statistical Analysis and Reporting** | **Final quality rating** |
| --- | --- | --- | --- | --- | --- | --- | --- |
| Shaya et al. 2021 | Moderate risk | High risk | High risk | Low risk | Moderate risk | Low risk | Moderate risk |
| Knutson et al. 2024 | Low risk | Low risk | Low risk | Low risk | Low risk | Low risk | Low risk |
| Bono et al. 2024 | Moderate risk | Moderate risk | Moderate risk | Low risk | Low risk | Moderate risk | Moderate risk |
| Conteduca et al. 2022 | Moderate risk | Moderate risk | Moderate risk | Low risk | Low risk | Moderate risk | Moderate risk |
| Annala et al. 2021 | Moderate risk | Moderate risk | Low risk | Low risk | Low risk | Moderate risk | Moderate risk |
| Goodall et al. 2020 | Moderate risk | Moderate risk | Low risk | Low risk | Low risk | Moderate risk | Moderate risk |
| Sweeney et al. 2024 | Low risk | Moderate risk | Low risk | Low risk | Low risk | Moderate risk | Moderate risk |
| Maurice-Dror et al. 2021 | Moderate risk | Moderate risk | Moderate risk | Low risk | Low risk | Moderate risk | Moderate risk |
| Tolmeijer et al. 2024 | Moderate risk | Moderate risk | Moderate risk | Low risk | Moderate risk | Moderate risk | Moderate risk |
| Kohli et al. 2020 | Moderate risk | Moderate risk | Moderate risk | Low risk | Low risk | Moderate risk | Moderate risk |
| Mizuno et al. 2021 | Moderate risk | Moderate risk | Moderate risk | Low risk | Low risk | Moderate risk | Moderate risk |
| Tolmeijer et al. 2023 | Moderate risk | Moderate risk | Low risk | Low risk | Low risk | Moderate risk | Moderate risk |
| Sumanasuriya et al. 2021 | Moderate risk | Moderate risk | Low risk | Low risk | Low risk | Moderate risk | Moderate risk |
| Torquato et al. 2019 | Moderate risk | Moderate risk | Moderate risk | Low risk | Low risk | Moderate risk | Moderate risk |
| Ruiz-Vico et al. 2024 | Moderate risk | Moderate risk | Low risk | Low risk | Low risk | Moderate risk | Moderate risk |
| Kwan et al. 2025 | Moderate risk | Moderate risk | Low risk | Low risk | Low risk | Moderate risk | Moderate risk |
| Khalaf et al. 2018 | Moderate risk | Moderate risk | Low risk | Low risk | Low risk | Moderate risk | Moderate risk |
| Stover et al. 2021 | Moderate risk | Moderate risk | Moderate risk | Low risk | Low risk | Moderate risk | Moderate risk |
| Jayaram et al. 2021 | Moderate risk | Moderate risk | Moderate risk | Low risk | Low risk | Moderate risk | Moderate risk |
| Fonseca et al. 2024 | Moderate risk | Moderate risk | Low risk | Low risk | Low risk | Moderate risk | Moderate risk |
| Nørgaard et al. 2023 | Moderate risk | Moderate risk | Low risk | Low risk | Low risk | Moderate risk | Moderate risk |
| Reichert et al. 2022 | Moderate risk | Low risk | Low risk | Low risk | Low risk | Moderate risk | Low risk |
| Pan et al. 2022 | Moderate risk | Moderate risk | Low risk | Low risk | Low risk | Moderate risk | Moderate risk |
| Azad et al. 2024 | Moderate risk | Low risk | Low risk | Low risk | Low risk | Moderate risk | Low risk |


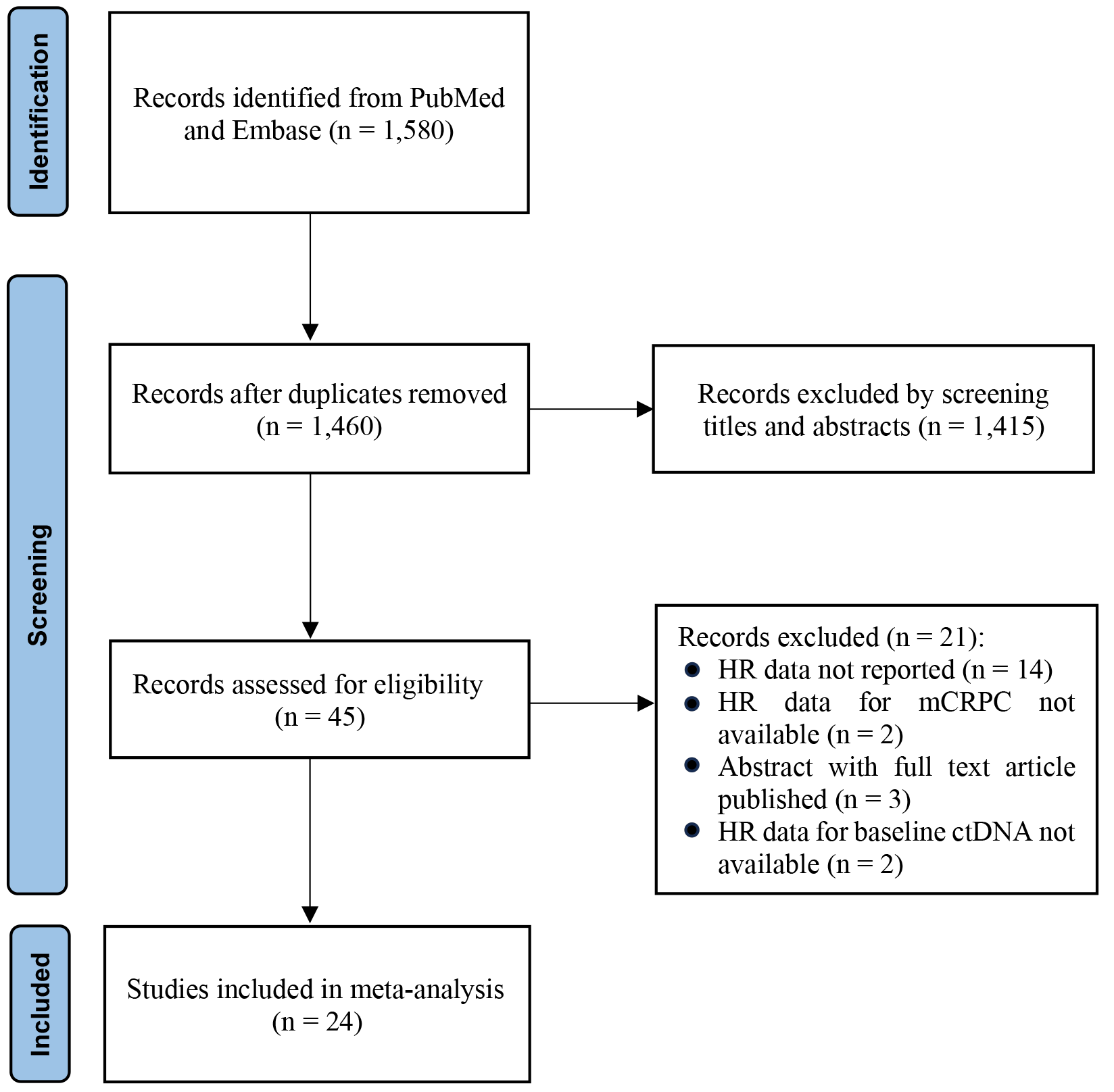


**Supplementary Figure 1.** PRISMA flow diagram of study selection.


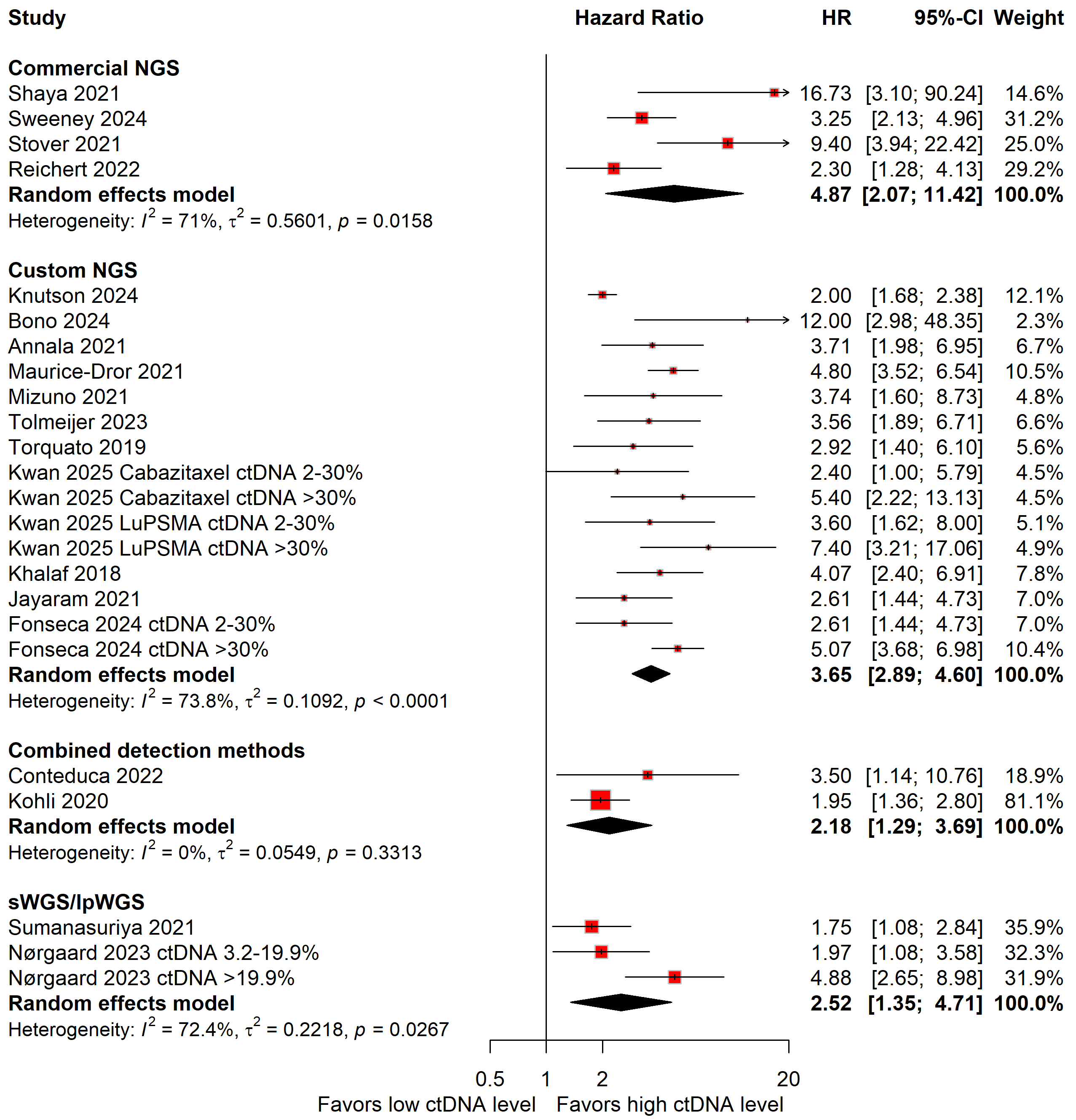


**Supplementary Figure 2.** Forest plots demonstrating the association between baseline circulating tumor DNA levels and overall survival in patients with metastatic castration-resistant prostate cancer stratified by detection method.


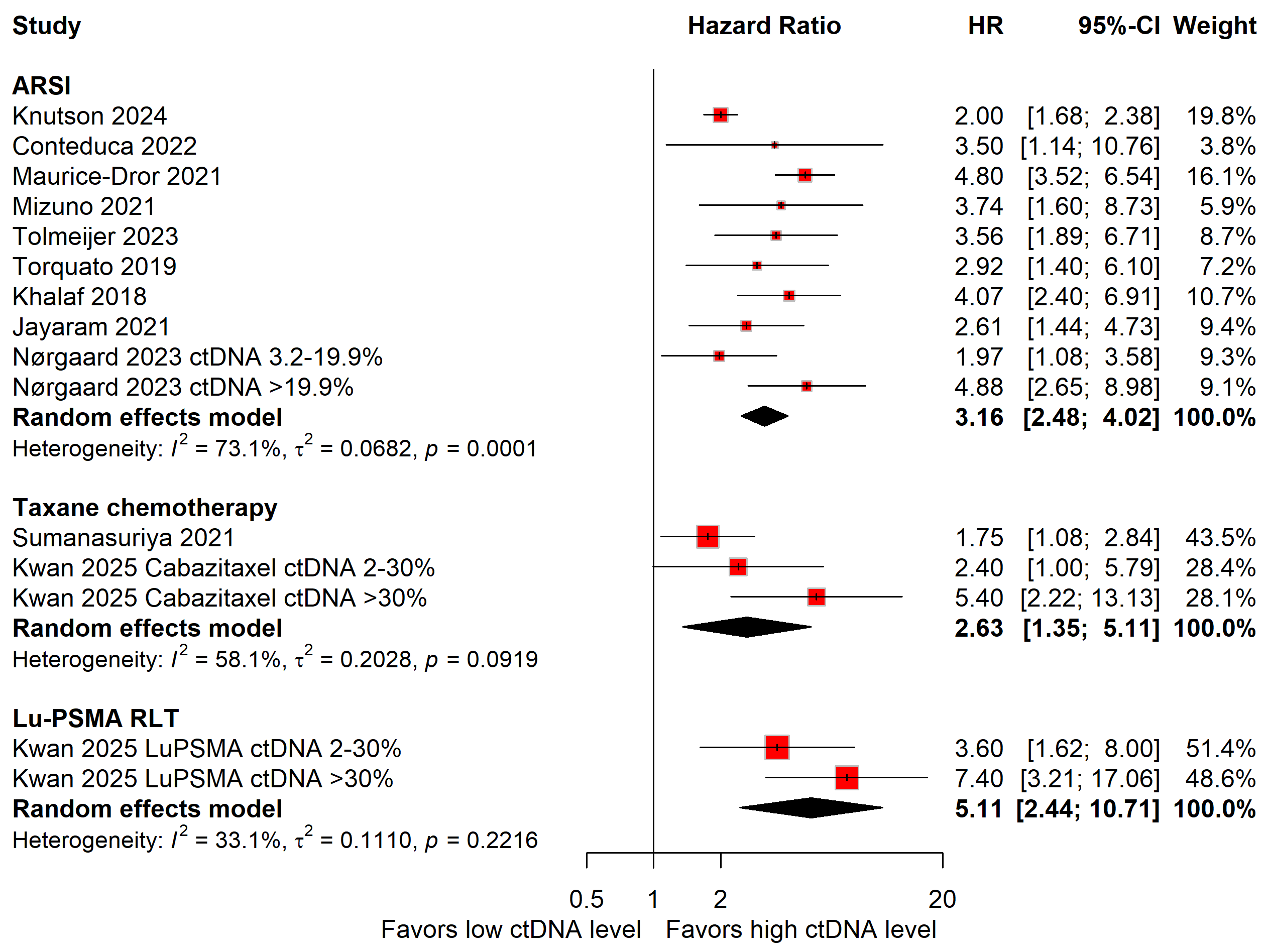


**Supplementary Figure 3.** Forest plots demonstrating the association between baseline circulating tumor DNA levels and overall survival in patients with metastatic castration-resistant prostate cancer stratified by treatment modality.


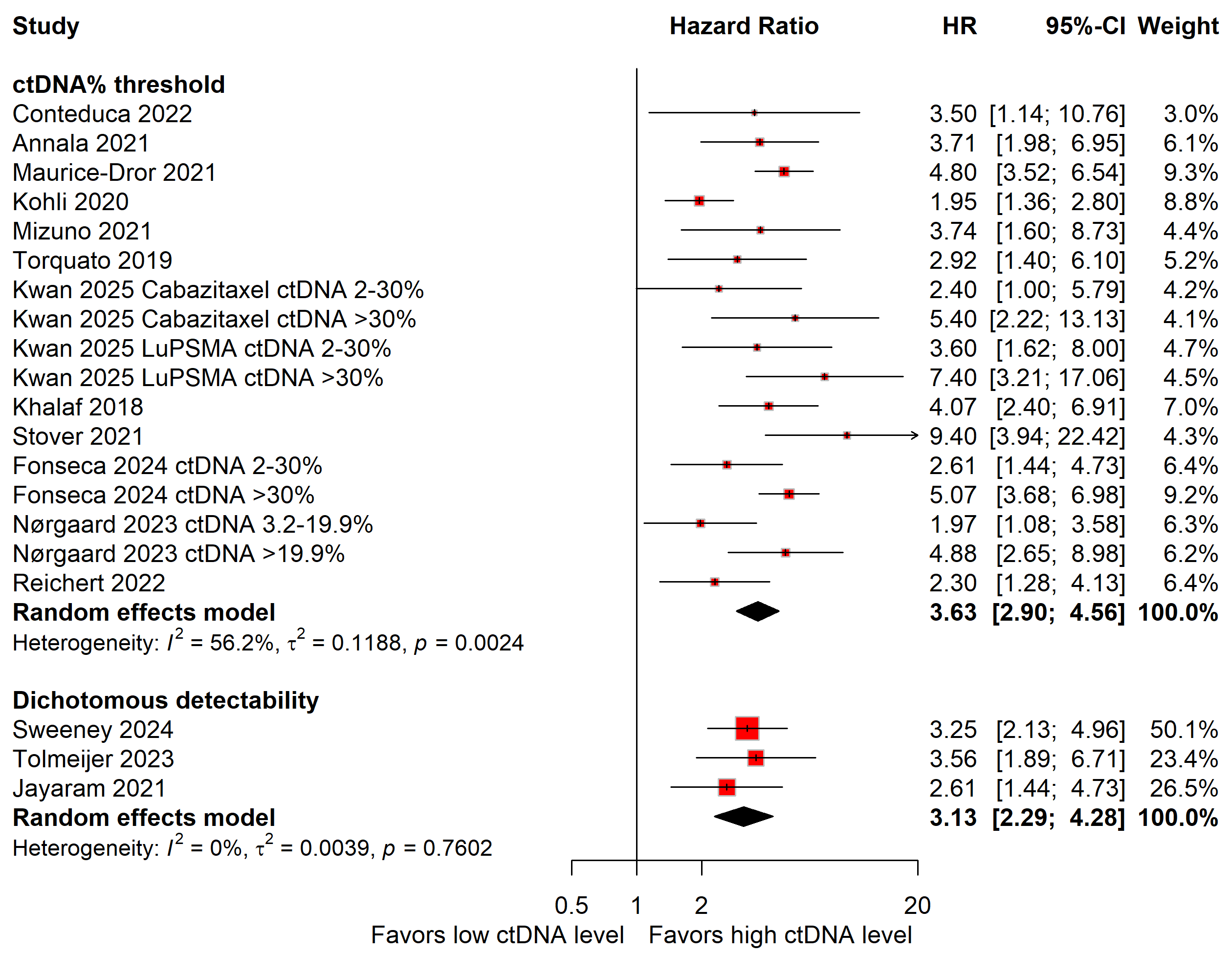


**Supplementary Figure 4.** Forest plots demonstrating the association between baseline circulating tumor DNA levels and overall survival in patients with metastatic castration-resistant prostate cancer stratified by definition of high/positive ctDNA.


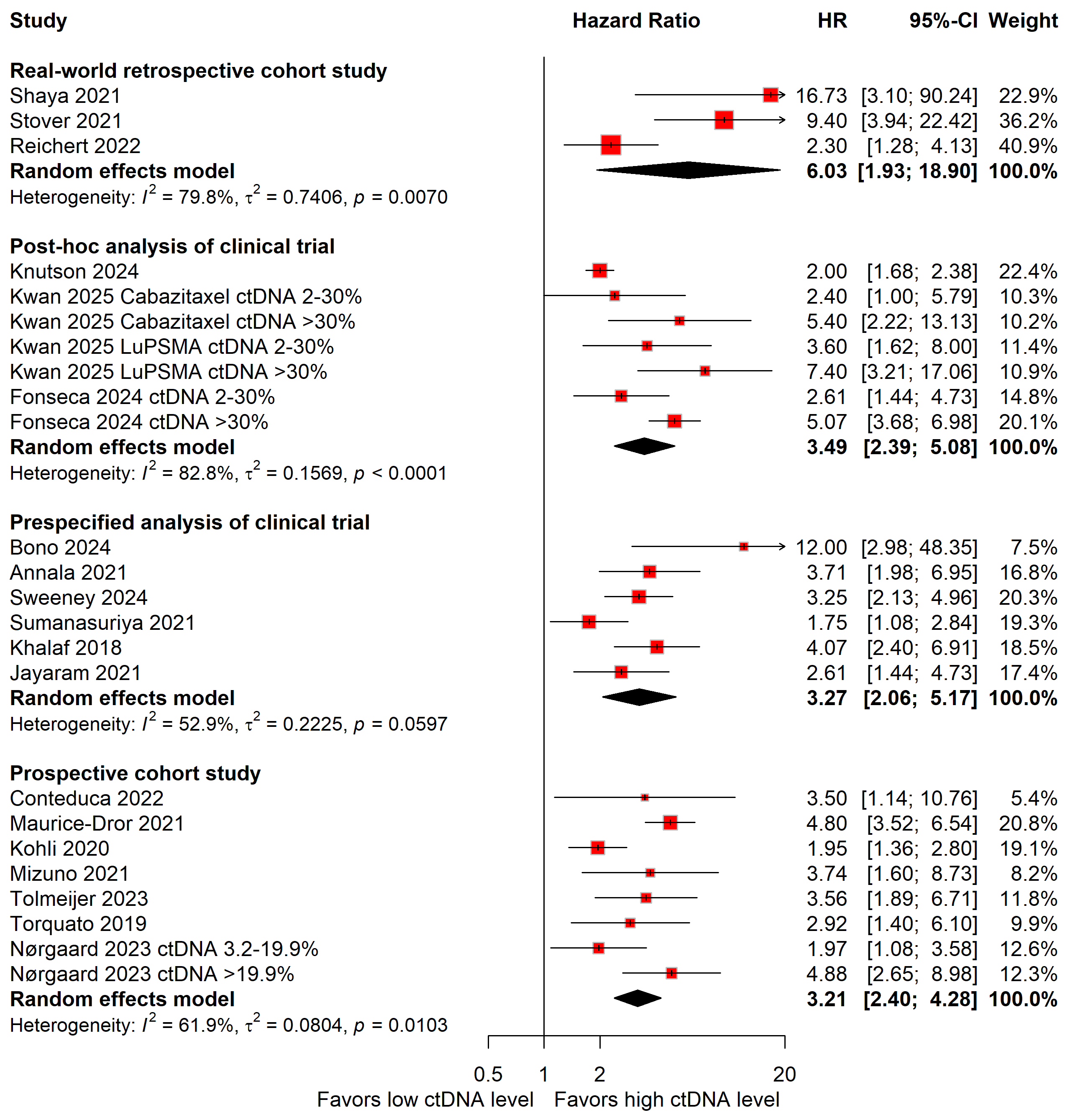


**Supplementary Figure 5.** Forest plots demonstrating the association between baseline circulating tumor DNA levels and overall survival in patients with metastatic castration-resistant prostate cancer stratified by study design.


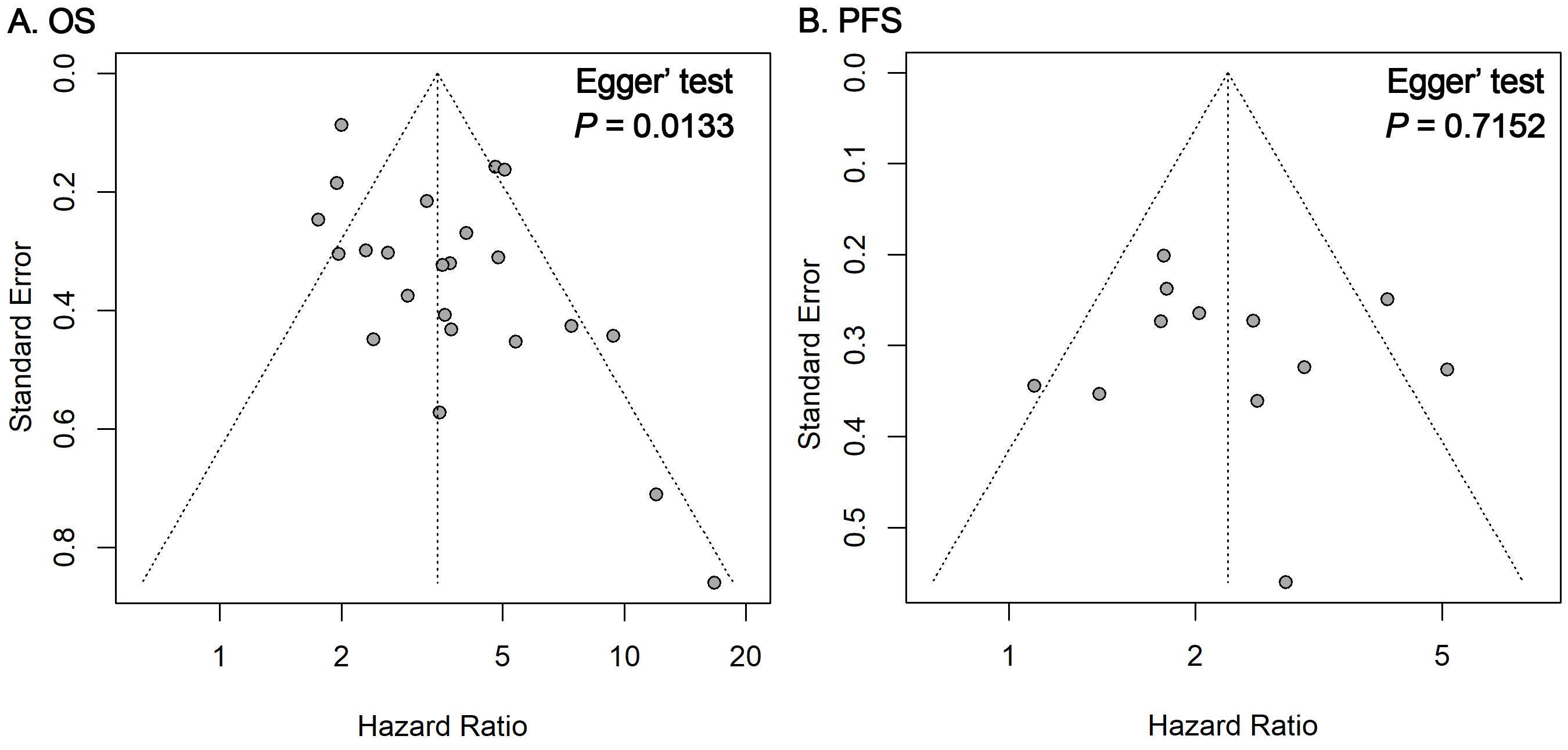


**Supplementary Figure 6.** Funnel plots and Egger’s tests for overall survival and progression-free survival.


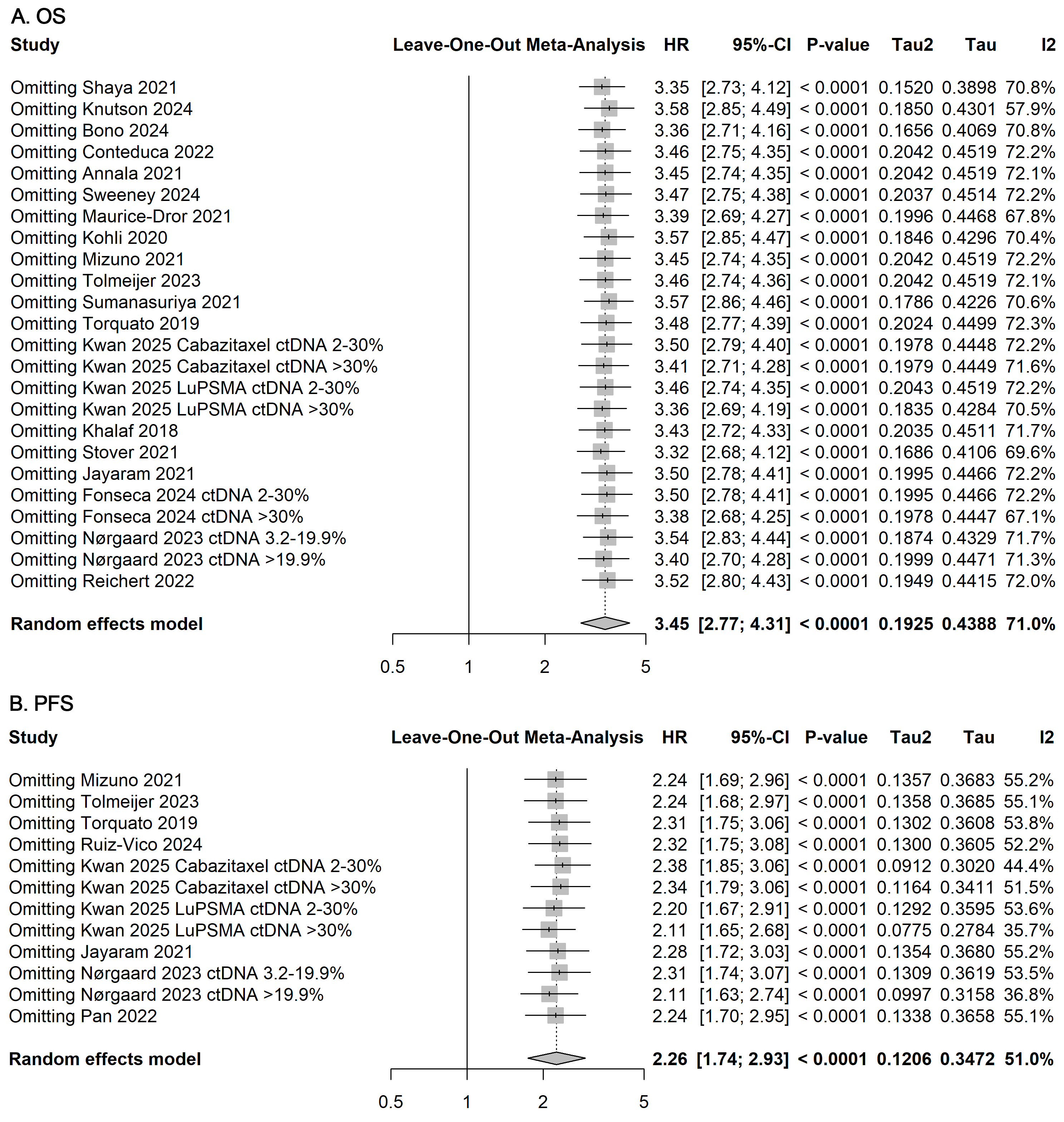


**Supplementary Figure 7.** Sensitivity analyses for included studies on overall survival and progression-free survival.


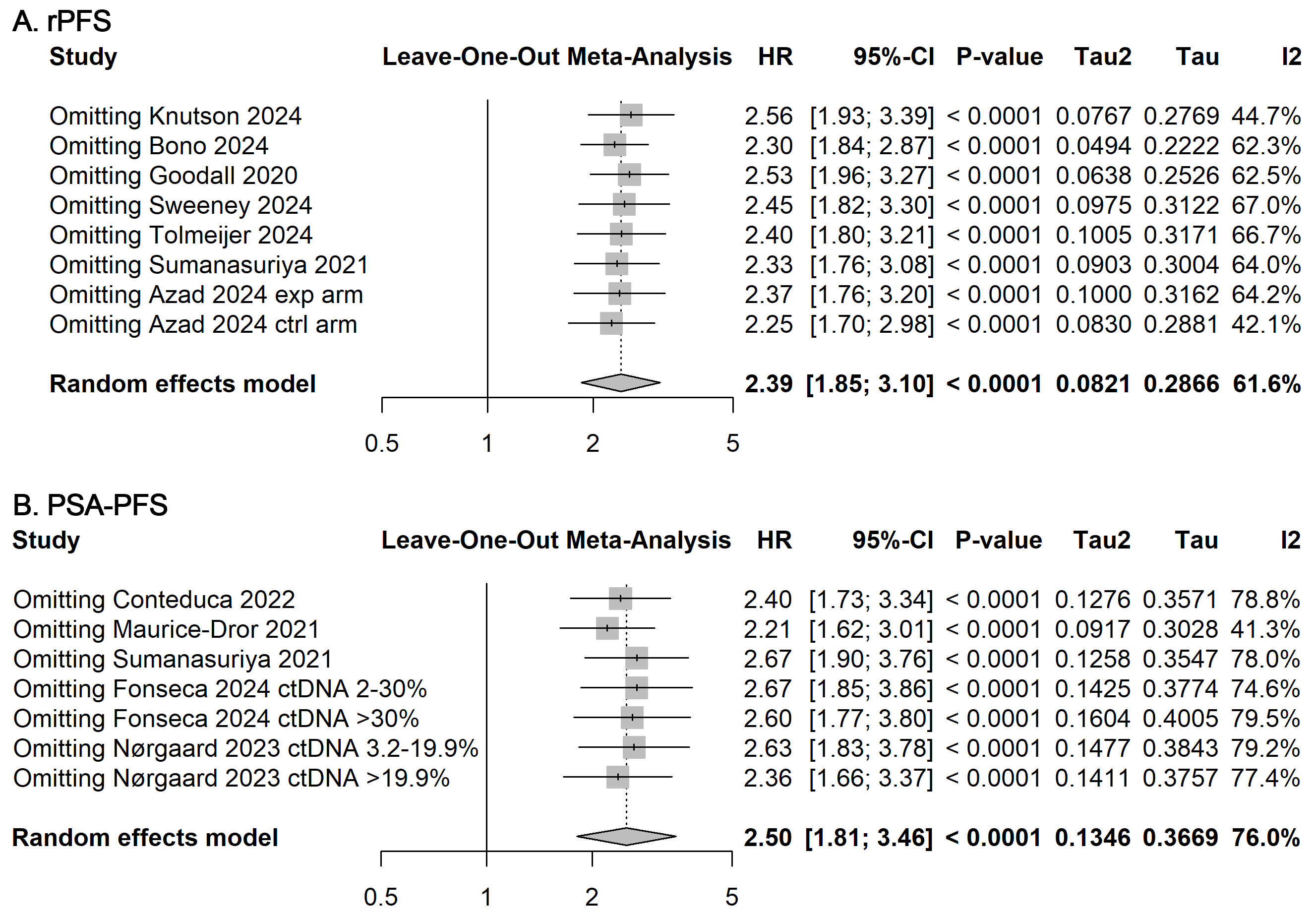


**Supplementary Figure 8.** Sensitivity analyses for included studies on radiographic progression-free survival and prostate specific antigen progression-free survival.
